# Personalizing the Prediction of Colorectal Cancer Prognosis by Incorporating Comorbidities and Functional Status into Prognostic Nomograms

**DOI:** 10.3390/cancers11101435

**Published:** 2019-09-26

**Authors:** Daniel Boakye, Lina Jansen, Martin Schneider, Jenny Chang-Claude, Michael Hoffmeister, Hermann Brenner

**Affiliations:** 1 Division of Clinical Epidemiology and Aging Research, German Cancer Research Center (DKFZ), 69120 Heidelberg, Germany; d.boakye@dkfz.de (D.B.);; 2 Medical Faculty Heidelberg, Heidelberg University, 69120 Heidelberg, Germany; 3 Department of General, Visceral and Transplantation Surgery, Heidelberg University Hospital, 69120 Heidelberg, Germany; 4 Unit of Genetic Epidemiology, Division of Cancer Epidemiology, German Cancer Research Center (DKFZ), 69120 Heidelberg, Germany; 5 Cancer Epidemiology Group, University Cancer Center Hamburg (UCCH), University Medical Center Hamburg-Eppendorf (UKE), 20251 Hamburg, Germany; 6 Division of Preventive Oncology, German Cancer Research Center (DKFZ) and National Center for Tumor Diseases (NCT), 69120 Heidelberg, Germany; 7 German Cancer Consortium (DKTK), German Cancer Research Center (DKFZ), 69120 Heidelberg, Germany

**Keywords:** comorbidity, functional status, nomogram, personalized medicine, prognosis, colorectal neoplasm

## Abstract

Despite consistent evidence that comorbidities and functional status (FS) are strong prognostic factors for colorectal cancer (CRC) patients, these important characteristics are not considered in prognostic nomograms. We assessed to what extent incorporating these characteristics into prognostic models enhances prediction of CRC prognosis. CRC patients diagnosed in 2003–2014 who were recruited into a population-based study in Germany and followed over a median time of 4.7 years were randomized into training (*n* = 1608) and validation sets (*n* = 1071). In the training set, Cox models with predefined variables (age, sex, stage, tumor location, comorbidity scores, and FS) were used to construct nomograms for relevant survival outcomes. The performance of the nomograms, compared to models without comorbidity and FS, was evaluated in the validation set using concordance index (C-index). The C-indexes of the nomograms for overall and disease-free survival in the validation set were 0.768 and 0.737, which were substantially higher than those of models including tumor stage only (0.707 and 0.701) or models including stage, age, sex, and tumor location (0.749 and 0.718). The nomograms enabled significant risk stratification within all stages including stage IV. Our study suggests that incorporating comorbidities and FS into prognostic nomograms could substantially enhance prediction of CRC prognosis.

## 1. Introduction

Colorectal cancer (CRC) is the second leading cause of cancer deaths in the world, accounting for almost 900,000 deaths annually [1]. Although increased use of screening and improved treatment strategies have led to better prognosis of CRC patients in many countries [2,3], about 40% of the patients still die within five years of diagnosis [3].

Given the strong dependency of CRC patients’ prognosis on stage at diagnosis, the TNM (tumor–node–metastasis) staging system is central for prognostication of CRC [4,5]. However, it exclusively takes tumor spread into account. Several nomograms were, therefore, proposed in the past decade to enhance prediction of CRC prognosis [6,7,8,9,10,11,12,13,14,15,16]. These nomograms included additional relevant variables and demonstrated higher predictive capacities than tumor stage, but mostly focused on tumor characteristics. In the era of personalized oncology, patients’ factors such as comorbidities and functional status often receive less attention as prognostic factors but could be equally important as tumor characteristics. Few nomograms incorporated functional status [9,10,11,14], but were derived from data on patients enrolled in randomized controlled trials (RCTs) who are often younger, have fewer comorbidities, and have higher functional status than unselected patient populations. In addition, these nomograms were designed for specific patient groups (e.g., stage III [9] and IV [14]) or for short-term outcomes only (six-month mortality) [11]. A recent, large population-based study from England [16] incorporated some specific comorbidities but did not consider functional status.

Given that CRC is mostly diagnosed in older adults, in whom comorbidities and functional impairment are common, we anticipate that adding these important patients’ characteristics to prognostic nomograms could enhance the individualized prediction of prognosis. In this population-based study of CRC patients, we aimed, for the first time, to develop nomograms for various survival outcomes by incorporating both comorbidities and functional status in addition to the commonly considered patient and tumor characteristics.

## 2. Methods

### 2.1. Patient Population

Our analyses were based on data on CRC patients who were diagnosed in 2003–2014 and recruited into the DACHS (Darmkrebs: Chancen der Verhütung durch Screening) study, a population-based case-control study with additional regular follow up of cases conducted in southern Germany. In brief, patients with first-time diagnosis of CRC (International Classification of Diseases, 10th Revision (ICD-10), codes C18–C20) and aged ≥30 years were eligible. Participants were recruited from all 22 hospitals providing first-line treatment for CRC in the study region. Data from cancer registries indicate that about half of the eligible patients in the study region of about two million inhabitants were recruited. Further details of the DACHS study were described elsewhere [17,18]. The DACHS study was approved by the ethics committees of the state medical boards of Baden-Wuerttemberg (M-198-02) and Rhineland-Palatinate (837.419.02 [3637]) and the Medical Faculty of Heidelberg University (310/2001). All participants gave written informed consent.

At baseline, trained interviewers conducted interviews with the participants to collect information on lifestyle factors and medical history, using a standardized questionnaire. Detailed medical information on tumor and patient characteristics, including comorbidities and functional status, was extracted from hospital records. Follow-up started from CRC diagnosis, and vital status and cause of death were ascertained from population registries and public health authorities about three, five, and 10 years after diagnosis. Information on newly diagnosed cancers and recurrence was additionally obtained from physicians around three, five, and 10 years after diagnosis. In patients who died during follow-up or were lost to follow-up, information on recurrence was obtained from the last attending physician.

### 2.2. Inclusion Criteria

Our analytic sample comprised all CRC patients recruited in hospitals that reported functional status data for ≥75% of the patients per recruitment year who underwent surgical resection of tumor and survived for ≥1 month (Figure S1). Patients with no information on tumor stage were excluded. A total of 2679 patients were included in our analyses and were randomized into training (*n* = 1608) and validation sets (*n* = 1071) in a ratio of 60:40.

### 2.3. Ascertainment of Comorbidities and Functional Status

We abstracted ICD-10 codes for various comorbidities that were diagnosed either prior to or at the time of CRC diagnosis from medical records (Table S1). An overall comorbidity score was then computed using the Charlson comorbidity index (CCI) [19]. We used restricted cubic spline functions to assess nonlinear association of CCI score with all-cause mortality and to choose clinically meaningful cut offs in the training set. Patients were grouped into four groups, namely, CCI score 0 (no comorbidity), 1–2, 3, or 4+ (very severe comorbidity). Perioperative assessment of functional status varied between hospitals and within hospitals over time. The most commonly used scales were the American Society of Anesthesiologists (ASA) grade [20], Eastern Cooperative Oncology Group (ECOG) score [21], and Karnofsky Performance Score (KPS) [22]. ASA is a five-grade (I–V) scale, with higher grades indicating poor functional status [20]. ECOG assesses functional status on a six-point scale (0–5), with higher scores indicating worse functional status [21]. KPS rates functional status on a scale of 0–100; a score of 100 indicates optimal health [22]. In the training set, we used the European Society for Medical Oncology (ESMO) criterion for comparing ECOG and KPS [21,23] and the regression coefficients of ASA, ECOG, and KPS for all-cause mortality to classify patients into three functional status groups—excellent (ASA = I–II, ECOG = 0, or KPS = 100), fair (ASA = III, ECOG = 1, or KPS = 70–90), and poor functional status (ASA = IV, ECOG = 2–4, or KPS = 10–60).

### 2.4. Selection of Variables

Factors that are consistently and strongly associated with CRC prognosis (e.g., hazard ratio ≥ 2.00 for all-cause mortality) independent of stage include age [11], comorbidities [24], and functional status [11,25,26]. Chemotherapy is also strongly associated with CRC prognosis but mostly in stage III/IV patients only [16]. Associations of lifestyle factors such as smoking and physical activity with CRC prognosis are only modest and most likely explained by comorbidity and functional status. We, therefore, selected age, comorbidities, functional status, and tumor stage a priori for our analyses, in addition to sex and tumor location. Our aim was to develop nomograms from parsimonious models that incorporate relevant and easily accessible variables only, as appropriate to prevent over-fitted models [27].

### 2.5. Outcomes

Our main outcomes were three- and five-year overall survival (OS; death from any cause) and disease-free survival (DFS; death from any cause or recurrence of CRC). Secondary outcomes investigated were disease-specific survival (DSS; mortality from CRC), recurrence-free survival (RFS; mortality from or recurrence of CRC), and non-disease-specific survival (mortality from causes other than CRC). Time was calculated from CRC diagnosis to the respective endpoints or end of follow-up, whichever occurred first.

### 2.6. Model Construction

Three separate Cox proportional hazards models with pre-specified variables for the various survival outcomes were trained in the training set. The basic model included tumor stage only (Union for International Cancer Control, I–IV) [5], the medium model additionally included age, sex, and tumor site, and the final model (used for the nomogram), furthermore, included CCI scores and functional status. We carefully assessed the proportional hazards assumption by adding time-dependent interaction terms to the covariates and checking whether their effects were statistically significant. We also assessed for interactions among the variables. We then used regression coefficients from the models to calculate nomogram points (0 to 100; higher points indicate worse prognosis) and to construct nomograms for the various outcomes. Our nomograms were constructed using established guidelines for developing prediction models [28].

### 2.7. Model Validation

We compared the nomograms’ predictive accuracy with the basic and medium models, using Harrell’s concordance index (C-index) in both the training and validation sets. For patients of all stages, we used restricted cubic spline functions and the Akaike information criterion value to choose appropriate cut offs for risk stratification of patients for OS in the training set. The cut-offs were then applied to the internal validation set. Patients were classified into four nomo-risk groups based on their nomogram points, namely, low (<50 points), moderate (50–99 points), high (100–139 points), and very high risk (140+ points). In the internal validation set, risk stratification for OS by the nomogram was additionally illustrated by Kaplan–Meier survival curves and log-rank tests for trend for the four nomo-risk groups among patients in all stages combined and for tertiles of nomo-risk groups in stage-specific analyses. We, furthermore, calibrated the nomograms in 200 bootstrapped samples, by plotting the observed survival probabilities estimated by the Kaplan–Meier method against the nomogram-predicted survival probabilities.

### 2.8. Net Benefit of Adding Comorbidity and Functional Status to the Nomograms

In the validation set, we quantified the incremental benefit of adding comorbidity and functional status to the nomograms by continuous net reclassification improvement (NRI) [29], using >20 or ≤20 nomogram points added by comorbidity and/or functional status (which corresponds to the presence of at least one comorbid condition or functional status being fair/poor) as a criterion for upgrade/downgrade of risk. NRI quantifies the net proportion of patients with and without the event of interest who are reclassified as higher and lower risk, respectively [29]. It is increasingly used in clinical research to estimate improvements in risk prediction [30].

Algorithms for constructing nomograms and for calculating NRI were developed with the R program (version 3.3.2.) [31], using the *rms* and *nricens* packages, respectively. All other analyses were conducted with the SAS software, version 9.4 (SAS Institute, Cary, NC, USA). Statistical tests were two-sided, with α = 0.05%.

## 3. Results

### 3.1. Characteristics of the Study Participants

A total of 1608 and 1071 patients were assigned to the training and validation sets, respectively (Table 1). In the training set, 60% were men, and the median age of the participants was 70 years. Nearly half of the patients (46%) had CCI = 1+ and poor functional status. The majority of the tumors were located in the colon (63%); 22%, 30%, 33%, and 14% were in stage I, II, III, and IV, respectively. During a median follow-up of 4.7 years, 598 (37%) deaths occurred. Characteristics of the validation set were similar to those of the training set.

### 3.2. Association of Patient and Tumor Characteristics with Survival Outcomes

Table 2 shows adjusted hazard ratios (HR) for the associations of patient and tumor characteristics with various survival outcomes in the training set. Older age, higher CCI scores, lower functional status, and advanced stage were consistently associated with substantially shorter OS, DFS, DSS, RFS, and nDSS. HRs for CCI scores 1–2, 3, and 4+, compared to CCI = 0, were 1.13, 1.84, and 2.39 (*p*_Trend_ < 0.001) for OS. For fair and poor functional status, compared to excellent functional status, HRs for OS were 1.42 and 2.52 (*p*_Trend_ < 0.001). The magnitudes of the associations of comorbidity and functional status for the other survival outcomes were similar to those for OS but were particularly pronounced for nDSS, while that of tumor stage for nDSS was modest.

### 3.3. Prognostic Nomograms

**Figure 1:**
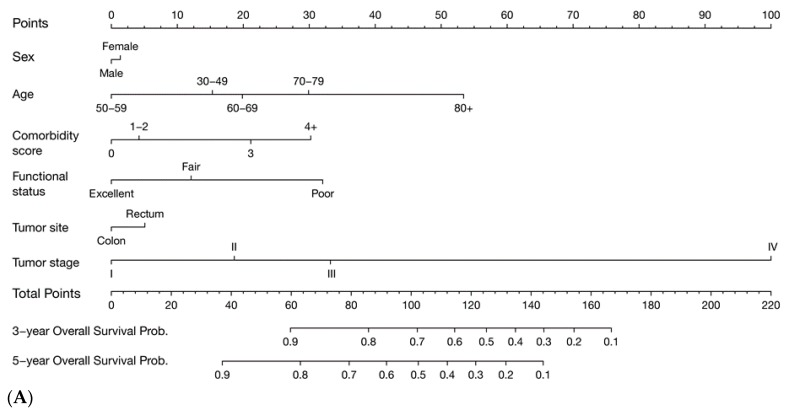

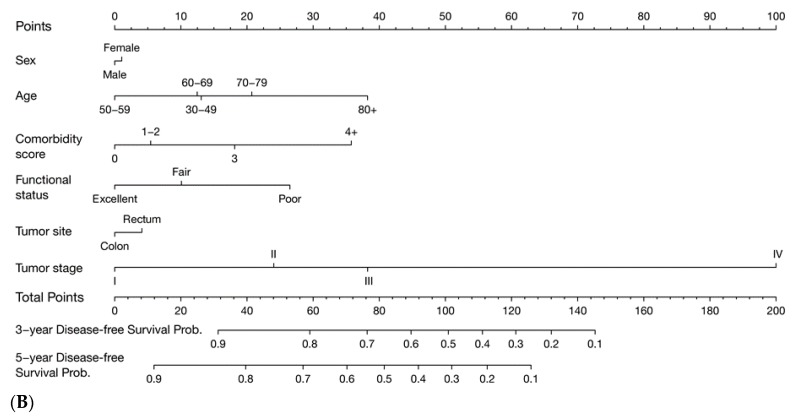
Nomograms for predicting three- and five-year overall and disease-free survival. Nomograms for (**A**) overall survival and (**B**) disease-free survival; Prob, probability. Total points were obtained by summing up individual points from the respective variables; higher points indicate poorer survival. Three- and five-year survival probabilities are then predicted using the total nomogram points.

Figure 1 shows the nomograms for OS and DFS, and Figures S2 and S3 show nomograms for the secondary outcomes. Tumor stage was the most important factor in all the nomograms, except for nDSS where age was the most important factor, followed by comorbidity and functional status, as reflected by the nomogram points. Comorbidity and functional status still contributed substantially to the prediction of CRC prognosis. Table S2 shows clinical examples of the application of the nomograms as an Excel calculator, demonstrating the important roles of comorbidity and functional status in the prediction of CRC prognosis. In the nomogram for OS, for example, a male patient aged 70 years, with stage III colon cancer, CCI = 0, and excellent functional status, will have total nomogram points of 63, corresponding to five-year OS of about 81%. In contrast, a patient with similar characteristics but with CCI = 4+ and poor functional status will have total nomogram points of 125, which equates to five-year OS of about 28%.

### 3.4. Validation of Nomograms

In all outcomes investigated, the nomograms outperformed the models with tumor stage only and those with age, sex, tumor location, and stage in both the training and internal validation sets (Table 3). In the validation set, the C-indexes (95% confidence interval) of the nomograms compared to models with stage only and those with age, sex, tumor location, and stage were 0.7680 (0.7426–0.7934), 0.7071 (0.6792–0.7350), and 0.7489 (0.7222–0.7755) for OS and 0.7369 (0.7112–0.7627), 0.7014 (0.6757–0.7271), and 0.7179 (0.6914–0.7445) for DFS, respectively. Addition of comorbidity and functional status improved the discrimination of the models for all outcomes and was particularly pronounced for OS, DFS, and nDSS and in stages I–III (C-indexes 0.706 vs. 0.672 for OS and 0.671 vs. 0.646 for DFS).

**Figure 2:**
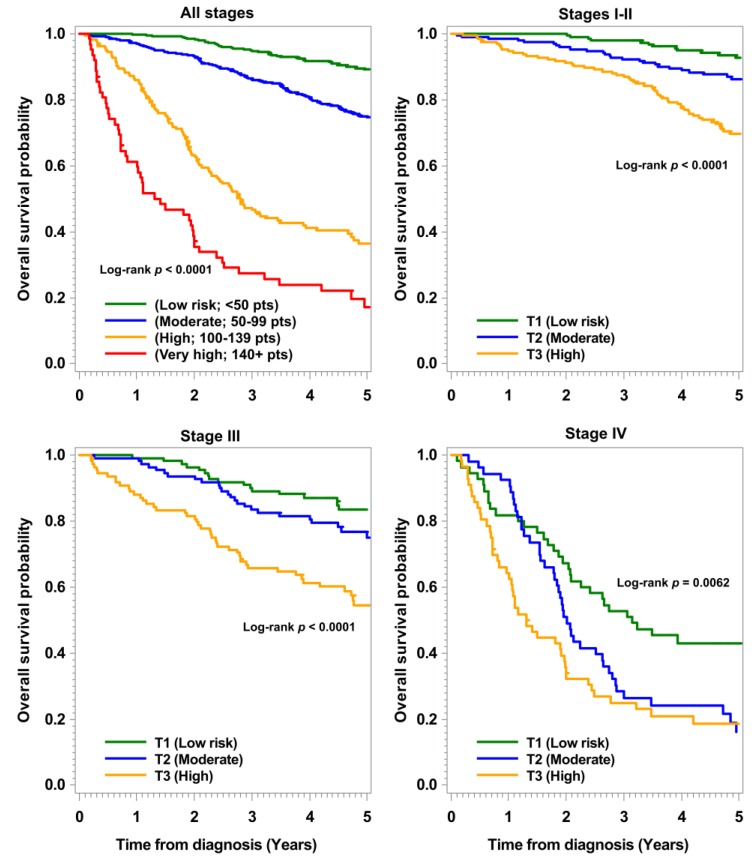
Overall survival stratified by nomogram risk groups in the validation set. T, tertile of total nomogram points.

Nomogram-defined risk groups showed strongly divergent survival probabilities across all stages (Figure 2). As shown in the calibration plots (Figure S4), the predicted three-year and five-year OS and DFS probabilities from the nomograms were close to the observed survival probabilities, indicating good reliability of the nomograms in an independent population. Near perfect calibrations were observed in the nomograms for DSS, RFS, and nDSS.

### 3.5. NRI of Adding Comorbidity and Functional Status to the Nomograms

We estimated the net benefit of adding comorbidities and functional status to the medium models, using continuous NRI in the validation set (Table 4). Results showed that addition of these characteristics to the models improved the classification of patients into lower and higher risk groups in all outcomes, even though improvements were statistically significant for OS, DFS, and DSS only. Stage-specific analyses showed that improvements were particularly pronounced for stages I–III.

## 4. Discussion

Patients’ characteristics such as comorbidities and functional status continue to receive less attention as prognostic factors but could be equally as important as tumor characteristics. We assessed to what extent incorporating comorbidities and functional status into prognostic nomograms enhances individualized prediction of CRC prognosis. We developed and validated nomograms for OS, DFS, DSS, RFS, and nDSS by adding comorbidity and functional status to models that included age, sex, tumor location, and stage, which had higher predictive accuracy than models without comorbidity and functional status.

Prognostication of CRC is dependent on the TNM system [4,5]. Despite its modifications, several limitations still exist. For instance, it focuses on tumor spread only [27], leading to heterogeneous survival rates in patients with the same stage [32]. Several nomograms were proposed to enhance prognostication of CRC [6,7,8,9,10,11,12,13,14,15,16,33]. These nomograms were consistently superior to tumor stage, but the studies from which they were constructed had limitations. Firstly, some used data on patients enrolled in clinical trials [10,11,14] and might, therefore, have limited validity in unselected patient populations. Secondly, most of the previous nomograms were restricted to specific patient groups (e.g., non-metastatic patients only) [6,9,10,33]. This could complicate patient care, as physicians would need different nomograms for different patient groups. Thirdly, despite consistent evidence that comorbidities [24] and functional status [25,26] are strong prognostic factors for CRC, these characteristics are not considered in nomograms. Most studies focused on tumor characteristics, with few incorporating additional blood-based markers such as carcinoembryonic antigen [6,12,13] and microRNA [33]. These markers are, however, not routinely collected in patient care, potentially limiting the use of these nomograms.

We developed nomograms that have high predictive accuracy, using few, but important, variables, which can easily be accessed from patients’ records. A nomogram built from variables that accurately predict patients’ outcomes could enhance clinical decision-making [27]. In our study, comorbidity and functional status were strong independent prognostic factors for CRC. Our nomograms incorporated these characteristics in addition to age, sex, tumor stage, and location, and demonstrated higher discriminatory accuracy than models including stage only or models with age, sex, tumor stage, and location. For example, the estimated five-year OS of a male patient aged 70 years, with stage III colon cancer, CCI = 0, and excellent functional status from our nomogram, was about 81%. In contrast, a male patient with similar characteristics but with CCI = 4+ and poor functional status is expected to have five-year OS of about 28%. In the United States (US) Surveillance, Epidemiology, and End Results (SEER) data, the estimated five-year OS for stage III patients is about 60% [32], but our nomogram suggests that patients with higher functional status and no comorbidity might have significantly higher survival than this average survival estimate, while those with lower functional status and severe comorbidities might have substantially lower survival. This demonstrates how important comorbidities and functional status are in the prediction of CRC prognosis aside from and on top of tumor characteristics. Diagnosis of CRC, like other cancer diagnoses, could lead to reduced adherence to treatment regimen and follow-up care for long-term conditions [34]. Comorbid patients might also be more likely to refuse and less likely to complete CRC treatments [35], even if they are deemed eligible for treatment by physicians or oncologists. Given the contribution of comorbidities and lower functional status to premature mortality and recurrence in CRC patients, medical care for CRC patients might benefit from more comprehensive, personalized consideration of both tumor and patient characteristics including comorbidities and functional status. The benefits of adding comorbidity and functional status to the prognostic tools were observed within all the tumor stages but were particularly pronounced in stages I–III. This is plausible given that the impact of comorbidity and functional status on CRC prognosis is stronger in early- than in advanced-stage tumors [24].

An important factor that should be considered when developing a nomogram is its ease of use. Although several nomograms for CRC were developed, little is known about their application in clinical care. A limitation of most of these nomograms is that they included many variables that either have less prognostic relevance or are not routinely collected in patient care. For instance, some nomograms incorporated variables like marital status [13], smoking [16], and body mass index [9,14,16], whose associations with CRC prognosis are only modest and most likely explained by comorbidity and functional status. We, thus, anticipate that including comorbidity and functional status in nomograms could act as surrogates for many factors, thereby reducing the number of variables required for prediction of CRC prognosis. Our nomograms and previous ones demonstrated higher predictive capacities than tumor stage alone, but ours have unique strengths. They incorporated few variables and still had very good discrimination even in the validation set (C-index for OS: 0.77), which was comparable to or even higher than those from studies that used many variables (C-index range: 0.75 to 0.79) [10,12,13,16]. This has merits, as physicians would need little and easily accessible information to use our nomograms, potentially enhancing their application.

Our nomograms were derived from data on patients recruited from 15 hospitals providing first-line treatment for CRC in the study region. This, together with the observed good discrimination and calibration of our nomograms in the internal validation set, suggests that our nomograms might be applicable to different populations. Aside from the nomograms for OS and DFS, we also developed prognostic tools for DSS, RFS, and nDSS to enhance risk stratification of patients for clinical trials. We were particularly able to quantify the incremental benefit of adding comorbidities and functional status to models that include age, sex, tumor location, and stage, using continuous NRI. Unlike Hippisley-Cox and Coupland who incorporated few comorbidities into their nomograms [16], we ascertained comorbidities in more detail and employed a standardized index [19] to quantify an overall comorbidity score.

Our study is, however, not without limitations. Because availability of functional status data varied between hospitals and within hospitals over time, we restricted our sample to patients recruited in hospitals that reported functional status data for ≥75% of the patients per recruitment year, in line with a predefined analytic protocol. The excluded sample had somewhat fewer comorbidities than the derivation set (45.1% vs. 49.0%), possibly because comorbidities were less comprehensively assessed in hospitals that recruited those patients. Also, we could not validate our nomograms in an external population, even though results from the internal validation set demonstrated very good predictive accuracy of the models. Further validation of our nomograms in external populations is, therefore, essential for establishing the generalizability of our findings. Lastly, patients with the same nomogram points could still have different survival rates because of inherent uncertainties in prediction models. For instance, we treated comorbidity and functional status as fixed variables, but these characteristics could change with tumor progression or treatment. The extent to which such changes might affect the accuracy of our models could not be estimated. Clinicians should, thus, take this into consideration when applying our nomograms.

## 5. Conclusions

Although evidence consistently shows that comorbidities and functional status are strong prognostic factors for CRC, these important characteristics are not considered in nomograms. We developed and validated nomograms for various survival outcomes by incorporating these characteristics in addition to age, sex, tumor location, and stage. Our study suggests that incorporating comorbidities and functional status into prognostic tools could enhance prediction of CRC prognosis.

## Figures and Tables

**Table 1:** Characteristics of colorectal cancer patients in both training and validation sets.

Characteristics	Total		Training Set		Validation Set	
	*n* = 2679		*n* = 1608		*n* = 1071	
	*n*	%	*n*	%	*n*	%
Sex						
Women	1051	39.2	639	39.7	412	38.5
Men	1628	60.8	969	60.3	659	61.5
Age (years)						
Median (Range)	70 (30–96)		70 (32–96)		70 (30–94)	
30–49	159	5.9	98	6.1	61	5.7
50–59	413	15.4	240	14.9	173	16.2
60–69	714	26.7	448	27.9	266	24.8
70–79	935	34.9	536	33.3	399	37.2
80+	458	17.1	286	17.8	172	16.1
Comorbidity score						
Median (Range)	0 (0–7)		0 (0–7)		0 (0–7)	
CCI 0	1451	54.2	871	54.2	580	54.2
CCI 1–2	901	33.6	531	33.0	370	34.5
CCI 3	179	6.7	107	6.6	72	6.7
CCI 4+	148	5.5	99	6.2	49	4.6
Functional status						
Excellent ^1^	1410	52.6	864	53.7	546	51.0
Fair ^2^	1131	42.2	662	41.2	469	43.8
Poor ^3^	138	5.2	82	5.1	56	5.2
Tumor location						
Colon	1685	62.9	1010	62.8	675	63.0
Rectum and rectosigmoid	994	37.1	598	37.2	396	37.0
Tumor stage, UICC						
I	594	22.2	361	22.4	233	21.8
II	832	31.0	485	30.2	347	32.4
III	862	32.2	535	33.3	327	30.5
IV	391	14.6	227	14.1	164	15.3
Follow up period (years)						
Median (IQR)	4.7 (3.3–5.3)		4.7 (3.3–5.3)		4.7 (3.3–5.3)	

ASA, American Society of Anesthesiologists (ASA) grade; CCI, Charlson comorbidity index score; ECOG, Eastern Cooperative Oncology (ECOG) score; IQR, interquartile range; KPS, Karnofsky performance score; UICC, Union for International Cancer Control staging system. ^1^ ASA I–II, ECOG 0, or KPS 100. ^2^ ASA III, ECOG 1, or KPS 70–90. ^3^ ASA IV, ECOG 2–4, or KPS 10–60.

**Table 2:** Association of patient and tumor characteristics with survival outcomes in the training set.

Variables	Primary Outcomes		Secondary Outcomes		
	OS	DFS ^†^	DSS	RFS ^†^	nDSS
	HR * (95% CI)	HR * (95% CI)	HR * (95% CI)	HR * (95% CI)	HR * (95% CI)
Sex					
Women	Ref	Ref	Ref	Ref	Ref
Men	0.96 (0.81–1.14)	0.97 (0.83–1.14)	**0.74 (0.59–0.92)**	**0.80 (0.65–0.97)**	**1.47 (1.12–1.92)**
Age					
30–49	1.56 (0.98–2.48)	1.42 (0.93–2.18)	1.31 (0.80–2.14)	1.32 (0.86–2.03)	1.58 (0.29–8.62)
50–59	Ref	Ref	Ref	Ref	Ref
60–69	**1.77 (1.26–2.49)**	**1.40 (1.04–1.89)**	1.34 (0.92–1.96)	1.16 (0.84–1.60)	**5.73 (2.06–15.95)**
70–79	**2.37 (1.70–3.30)**	**1.75 (1.31–2.35)**	**1.83 (1.26–2.64)**	1.37 (0.99–1.88)	**7.86 (2.85–21.66)**
80+	**4.66 (3.27–6.63)**	**2.81 (2.05–3.85)**	**2.61 (1.71–3.99)**	**1.60 (1.11–2.32)**	**19.68 (7.10–54.59)**
Comorbidity score					
CCI 0	Ref	Ref	Ref	Ref	Ref
CCI 1–2	1.13 (0.93–1.36)	1.16 (0.97–1.39)	0.99 (0.77–1.27)	1.03 (0.82–1.29)	**1.37 (1.02–1.86)**
CCI 3	**1.84 (1.36–2.49)**	**1.63 (1.21–2.21)**	**1.75 (1.15–2.66)**	1.28 (0.85–1.94)	**2.03 (1.30–3.17)**
CCI 4+	**2.39 (1.78–3.21)**	**2.63 (1.97–3.51)**	1.54 (0.97–2.46)	**1.80 (1.20–2.70)**	**3.81 (2.55–5.70)**
Functional status					
Excellent	Ref	Ref	Ref	Ref	Ref
Fair	**1.42 (1.17–1.72)**	**1.31 (1.10–1.57)**	**1.38 (1.09–1.76)**	**1.25 (1.01–1.56)**	**1.44 (1.06–1.97)**
Poor	**2.52 (1.82–3.48)**	**2.05 (1.49–2.82)**	**1.84 (1.14–2.97)**	**1.67 (1.09–2.56)**	**3.20 (2.02–5.05)**
Tumor location					
Colon	Ref	Ref	Ref	Ref	Ref
Rectum	1.16 (0.98–1.37)	1.12 (0.95–1.32)	1.10 (0.88–1.38)	1.06 (0.87–1.29)	1.10 (0.84–1.43)
Tumor stage					
I	Ref	Ref	Ref	Ref	Ref
II	**1.71 (1.27–2.31)**	**1.92 (1.44–2.55)**	**3.11 (1.50–6.46)**	**3.83 (2.16–6.82)**	**1.45 (1.04–2.02)**
III	**2.60 (1.96–3.46)**	**2.81 (2.13–3.71)**	**11.54 (5.87–22.72)**	**9.39 (5.43–16.24)**	1.08 (0.76–1.54)
IV	**17.84 (13.20–24.12)**	**14.94 (11.15–20.04)**	**92.15 (46.69–181.9)**	**50.30 (28.99–87.29)**	**1.99 (1.08–3.66)**

CCI, Charlson comorbidity index score; CI, confidence interval; DFS, disease-free survival; DSS, disease-specific survival; HR, hazard ratio; nDSS; non-disease-specific survival; OS, overall survival; Ref, reference; RFS, recurrence-free survival. ^†^ Thirty patients were excluded because they had recurrence or metastasis before the interview date. * Estimates were obtained from Cox regression and included all variables listed in the table; values in bold show statistically significant results.

**Table 3:** Comparison of concordance C-indexes of various prediction models, overall and according to tumor stage.

Survival Outcomes	Training Set			Validation Set		
	Harrell’s C-Index			Harrell’s C-Index		
	Stage Only	Model 2 ^†^	Nomogram ^‡^	Stage Only	Model 2 ^†^	Nomogram ^‡^
All patients						
Overall survival	0.7121	0.7745	0.7929	0.7071	0.7489	0.7680
Disease-free survival	0.7027	0.7333	0.7506	0.7014	0.7179	0.7369
Disease-specific	0.8199	0.8446	0.8498	0.8063	0.8229	0.8302
Recurrence-free survival	0.7749	0.7878	0.7920	0.7591	0.7678	0.7720
Non-disease-specific	0.5542	0.7504	0.7965	0.5240	0.7087	0.7503
Stages I–III						
Overall survival	0.6315	0.7254	0.7546	0.6124	0.6716	0.7062
Disease-free survival	0.6409	0.6836	0.7089	0.6215	0.6463	0.6713
Disease-specific	0.7557	0.7867	0.7988	0.7278	0.7478	0.7627
Recurrence-free survival	0.7297	0.7361	0.7445	0.6869	0.6946	0.7044
Non-disease-specific	0.5669	0.7554	0.8005	0.5246	0.7103	0.7492
Stage IV						
Overall survival	-	0.6207	0.6325	-	0.6018	0.6166
Disease-free survival	-	0.5982	0.6067	-	0.5822	0.5957
Disease-specific	-	0.6234	0.6377	-	0.5963	0.6136
Recurrence-free survival	-	0.6060	0.6113	-	0.5767	0.5947
Non-disease-specific	-	0.7983	0.8182	-	0.5573*	0.6193 *

C-index, concordance index. * Only eight (7.5%) patients died from causes other than colorectal cancer. ^†^ Stage, age, sex, and tumor location. ^‡^ Stage, age, sex, tumor location, comorbidity scores, and functional status.

**Table 4:** Net reclassification improvement of adding comorbidity and functional status to the nomogram, overall and stratified by tumor stage.

Outcomes	Validation Set		
	NRI_e_	NRI_ne_	NRI ^>0^ (95% CI) *
All patients			
Overall survival	−0.263	0.415	**0.152 (0.020–0.497)**
Disease-free survival	−0.297	0.513	**0.216 (0.070–0.336)**
Disease specific survival	−0.338	0.588	**0.250 (0.122–0.395)**
Recurrence-free survival	−0.580	0.657	0.077 (−0.031–0.293)
Non-disease-specific survival	−0.287	0.489	0.202 (−0.209–0.689)
Stages I–III			
Overall survival	0.248	0.386	**0.634 (0.224–0.787)**
Disease-free survival	−0.160	0.487	**0.327 (0.163–0.578)**
Disease specific survival	−0.320	0.544	0.224 (−0.423–0.607)
Recurrence-free survival	−0.116	0.563	**0.447 (0.082–0.691)**
Non-disease-specific survival	−0.350	0.618	0.222 (−0.066–0.932)
Stage IV			
Overall survival	0.284	0.101	**0.386 (0.012–0.738)**
Disease-free survival	−0.117	0.569	0.452 (−0.196–0.719)
Disease specific survival	−0.151	0.566	0.415 (−0.335–0.631)
Recurrence-free survival	−0.016	0.236	0.220 (−0.164–0.588)
Non-disease-specific survival	0.220	0.353	0.573 (−1.124; 1.803)

CI, confidence interval; e, events; NRI, net reclassification improvement; ne, non-events. NRI was calculated per increase in 20 nomogram points added by comorbidity and/or functional status. * Confidence intervals were calculated from 200 bootstrapped samples; estimates significantly different from 0 are highlighted in bold.

## Supplementary Materials

The following are available online at https://www.mdpi.com/2072-6694/11/10/1435/s1: Figure S1: Flow diagram showing selection of analytic sample, Figure S2: Nomograms for predicting three- and five-year disease-specific (A) and recurrence-free (B) survival, Figure S3: Nomogram for predicting three- and five-year mortality from causes other than colorectal cancer (non-disease-specific survival), Figure S4: Calibration plots for the prediction models in the validation set, Table S1: Definition and frequency of comorbidities in the Charlson comorbidity index (whole sample, *n* = 2679), Table S2: A calculator for estimating survival probabilities and showing the roles of comorbidities and functional status in the prediction of colorectal cancer prognosis.
