# Supplementary material for: Personalizing the Prediction of Colorectal Cancer Prognosis by Incorporating Comorbidities and Functional Status into Prognostic Nomograms
Source: Cancers (Basel). 2019 Sep 26;11(10):1435. doi: 10.3390/cancers11101435 (PMC6826360; doi:10.3390/cancers11101435)
Supplement: Supplementary file 1 [file cancers-11-01435-s001.zip › cancers-576682-suppl-layout.docx]

Supplementary Materials: Personalizing the Prediction of Colorectal Cancer Prognosis by Incorporating Comorbidities and Functional Status into Prognostic Nomograms

Daniel Boakye, Lina Jansen, Martin Schneider, Jenny Chang-Claude, Michael Hoffmeister and Hermann Brenner


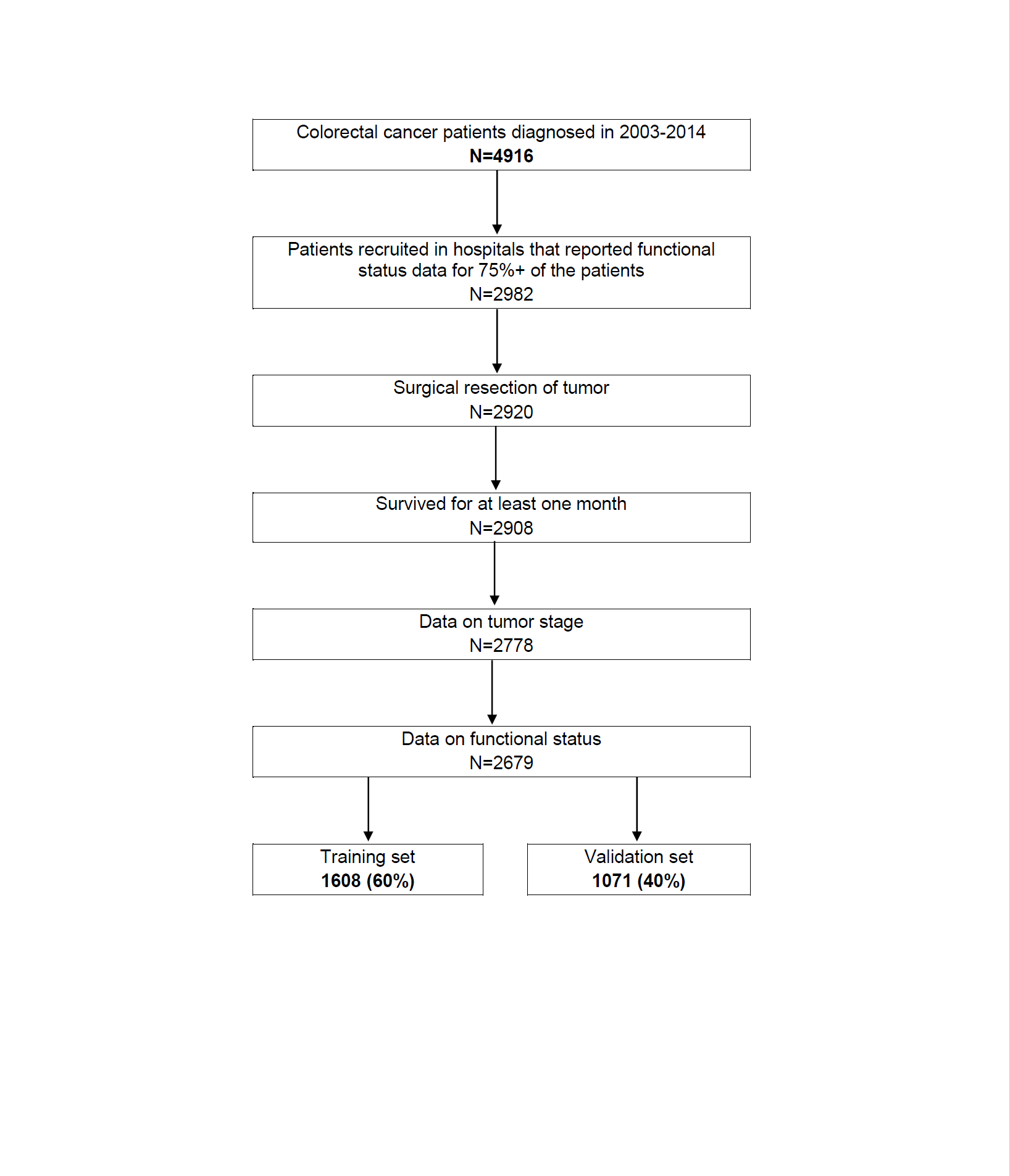


**Figure S1.** Flow diagram showing selection of analytic sample.


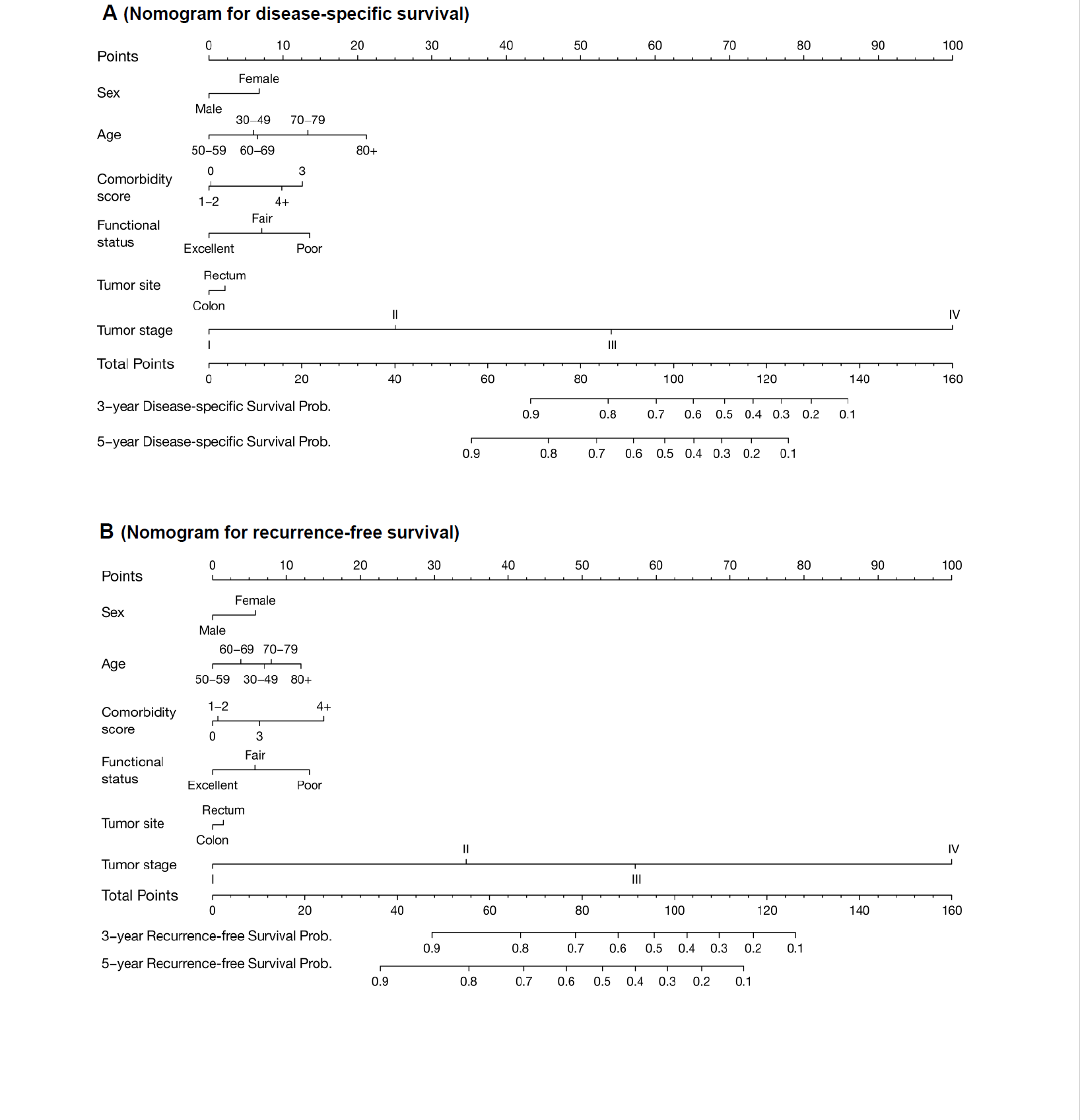


**Figure S2.** Nomograms for predicting 3- and 5-year disease-specific (**A**) and recurrence-free (**B**) survival. Prob, Probability. Total points were obtained by summing up individual points from the respective variables; higher points indicate poorer survival. Three- and five-year survival probabilities are then predicted using the total points.


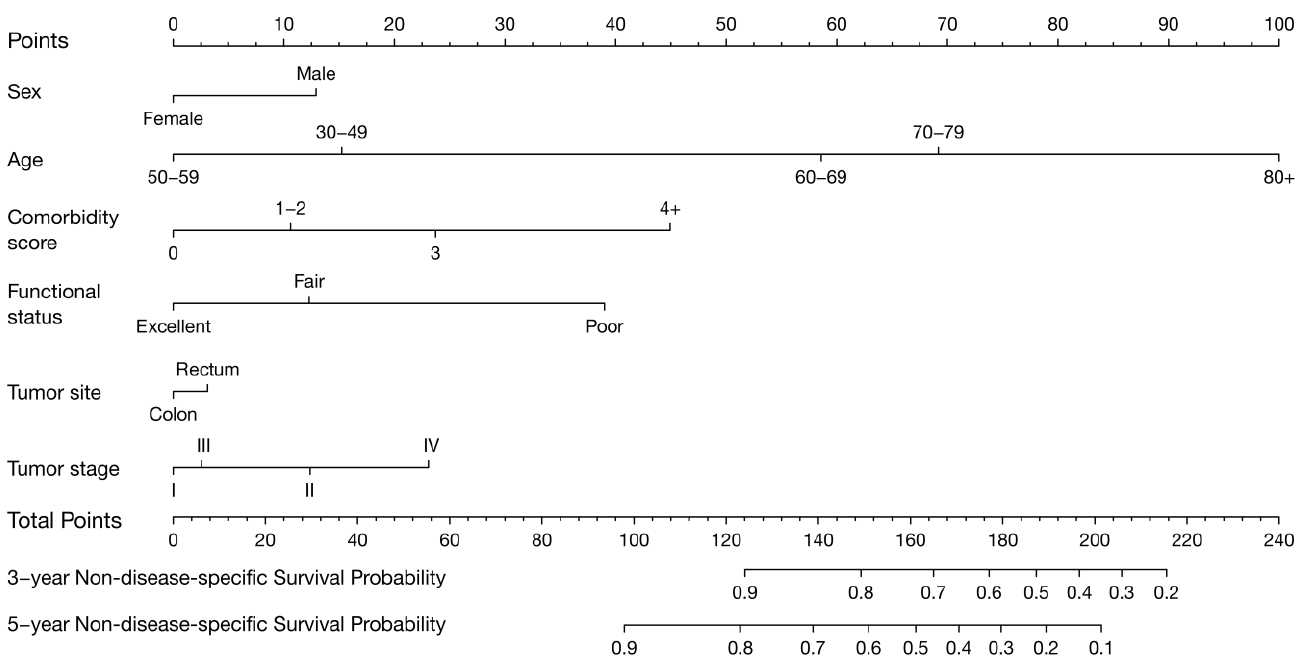


**Figure S3.** Nomogram for predicting 3- and 5-year mortality from causes other than colorectal cancer (non-disease-specific survival). Total points were obtained by summing up individual points from the respective variables; higher points indicate poorer survival. Three- and five-year survival probabilities are then predicted using the total points.


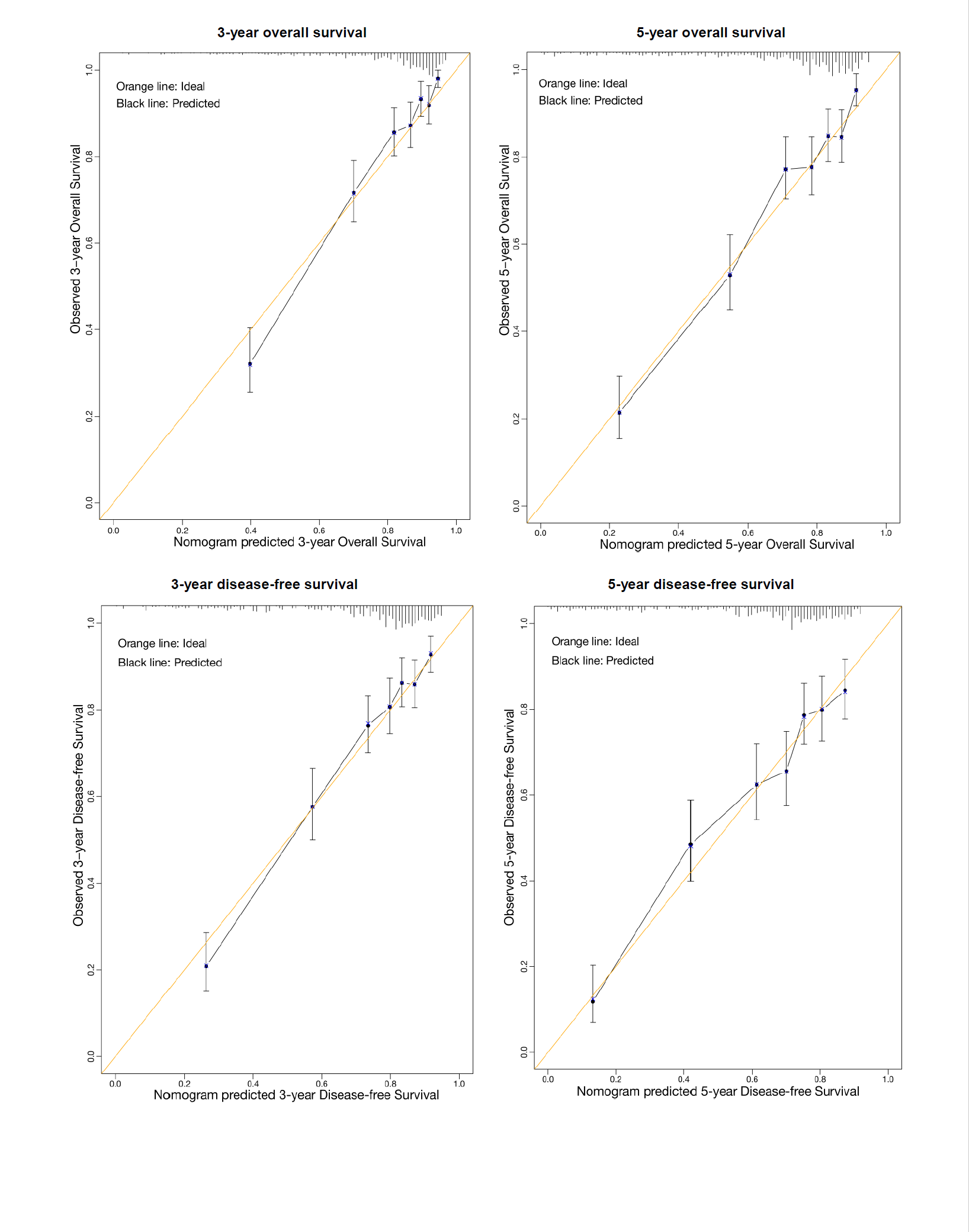


**Figure S4.** Calibration plots for the prediction models in the validation set, Table S1: Definition and frequency of comorbidities in the Charlson comorbidity index (whole sample, *n* = 2679). The horizontal bars represent 95% confidence intervals of the nomogram predicted survival probabilities and were calculated from 200 bootstrapped samples.

**Table S1.** Definition and frequency of comorbidities in the Charlson comorbidity index (whole sample, *n* = 2679).

| Comorbidities | ICD-10 Codes Used | n | % | Charlson’s Weight |
| --- | --- | --- | --- | --- |
| Myocardial Infarction | I21.1, I21.3, I21.4, I21.9, I22, I23, I25.1, I25.2, I25.6 | 215 | 8.0 | 1 |
| Congestive Heart Failure | I11.0, I13.0, I13.2, I50, I50.0, I50.1, I50.3, I50.4, I50.9 | 101 | 3.8 | 1 |
| Peripheral Vascular Disease | I70, I71, I72, I73, I77, K55 | 170 | 6.4 | 1 |
| Stroke & Hemi/Paraplegia | - | 147 | 5.5 |  |
| Stroke ^1^ | G45, G46, I60–I69, Z86.7 | *137* |  | 1 |
| Hemi/Paraplegia ^1^ | G11.4, G80, G81, G82, G83, I69.05 | *10* |  | 2 |
| COPD | J40–J47, J60–J70, J84.1, J92, J96.1, 98.2, J98.3, I27.8, I27.9 | 211 | 7.9 | 1 |
| Chronic Renal Disease | N01–N05, N07, N11, N17–N19, N25, I12.0, I13.1, I13.2, Z49, Z94.0, Z99.2 | 178 | 6.6 | 2 |
| Chronic liver disease | - | 150 | 5.6 |  |
| Mild ^1^ | K70 – K76, B18 | *149* | - | 1 |
| Moderate-Severe ^1^ | K70.4, K71.1, K72.1, K72.9, K76.7, B15, B16, B19, Z94.4 | *1* | - | 3 |
| Diabetes Mellitus | - | 474 | 17.7 |  |
| Without Complications ^1^ | E10.x–E14.x (where x = 0, 1 or 9) | *451* | - | 1 |
| With Complications ^1^ | E10.x–E14.x (where x = 2, 3, 4, 5, 6, 7 or 8) | *23* | - | 2 |
| Gastric/Duodenal Ulcer | K25–K28 | 15 | 0.6 | 1 |
| Rheumatoid Disease | M05, M06, M08, M30–M36 | 19 | 0.7 | 1 |
| Dementia | F00, F01, F02, F03, F05.1, G30, G30.9, G31.1 | 17 | 0.6 | 1 |
| Non-Colorectal Cancer (Except Basal Skin Carcinoma) | C00–C17, C21–C26, C30–C34, C37–C41, C43, C45–C58, C60–C76, C81–C85, C88, C90–C97, Z85.x (where x = 0, 2, 3, 4, 5, 6 or 8) | 272 | 10.2 | 2 |
| Charlson comorbidity index (CCI) score * | |  |  |  |
| Median (Range) | - | 0 (0–8) | - | - |
| CCI 0 (None) | - | 1451 | 54.2 | - |
| CCI 1–2 | - | 871 | 32.5 | - |
| CCI 3 | - | 189 | 7.0 | - |
| CCI 4+ (very severe) | - | 168 | 6.3 | - |

ICD, International Statistical Classification of Diseases; COPD, Chronic obstructive pulmonary disease. All primary tumors other than colorectal cancer (e.g. leukemia, lymphoma) were grouped under “non-colorectal cancer”. * HIV/AIDS did not contribute to the comorbidity scoring because none of the patients had this comorbid condition. ^1^ Are mutually exclusive (when both were present, the one with higher Charlson’s weight was considered).

| 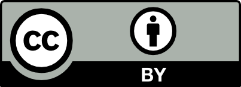 | © 2019 by the authors. Licensee MDPI, Basel, Switzerland. This article is an open access article distributed under the terms and conditions of the Creative Commons Attribution (CC BY) license (http://creativecommons.org/licenses/by/4.0/). |
| --- | --- |
